# Supplementary figures and images for: Cross-sectional white matter microstructure differences in age and trait mindfulness
Source: PLoS One. 2018 Oct 15;13(10):e0205718. doi: 10.1371/journal.pone.0205718 (PMC6188777; doi:10.1371/journal.pone.0205718)

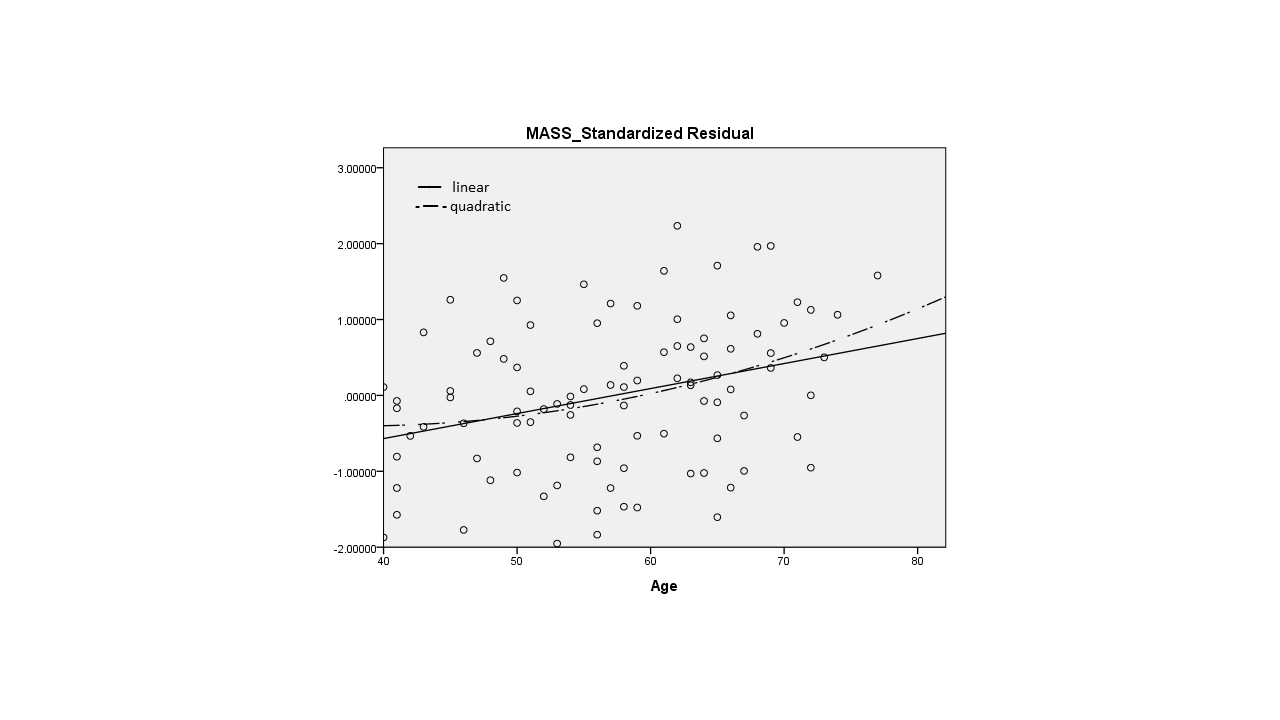

Supplement: S1 Fig — (TIF) [file pone.0205718.s001.tif]

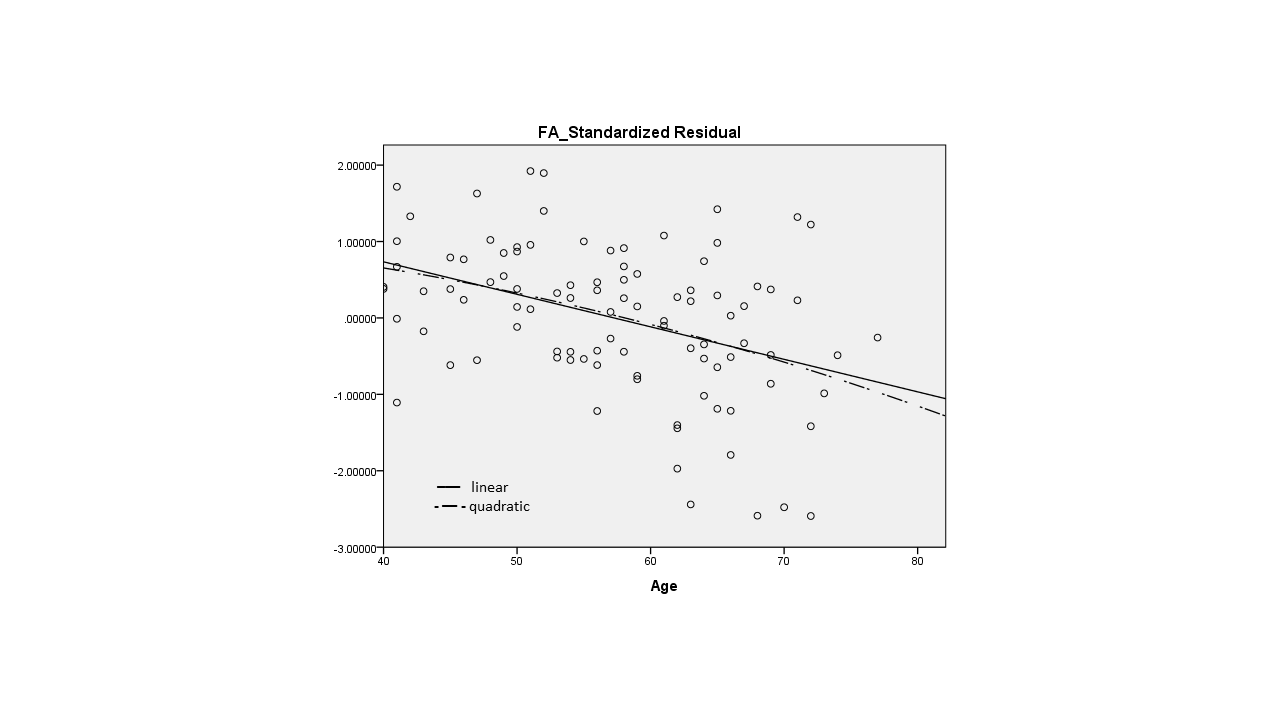

Supplement: S2 Fig — (TIF) [file pone.0205718.s002.tif]

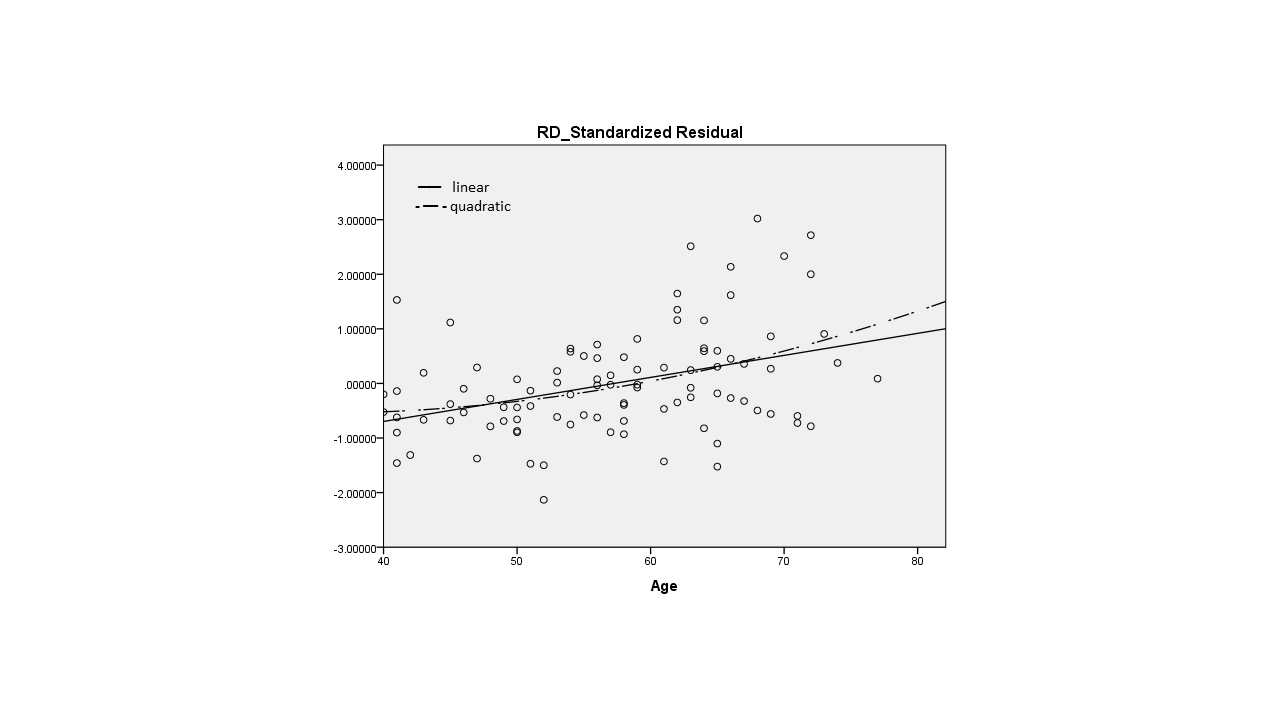

Supplement: S3 Fig — (TIF) [file pone.0205718.s003.tif]

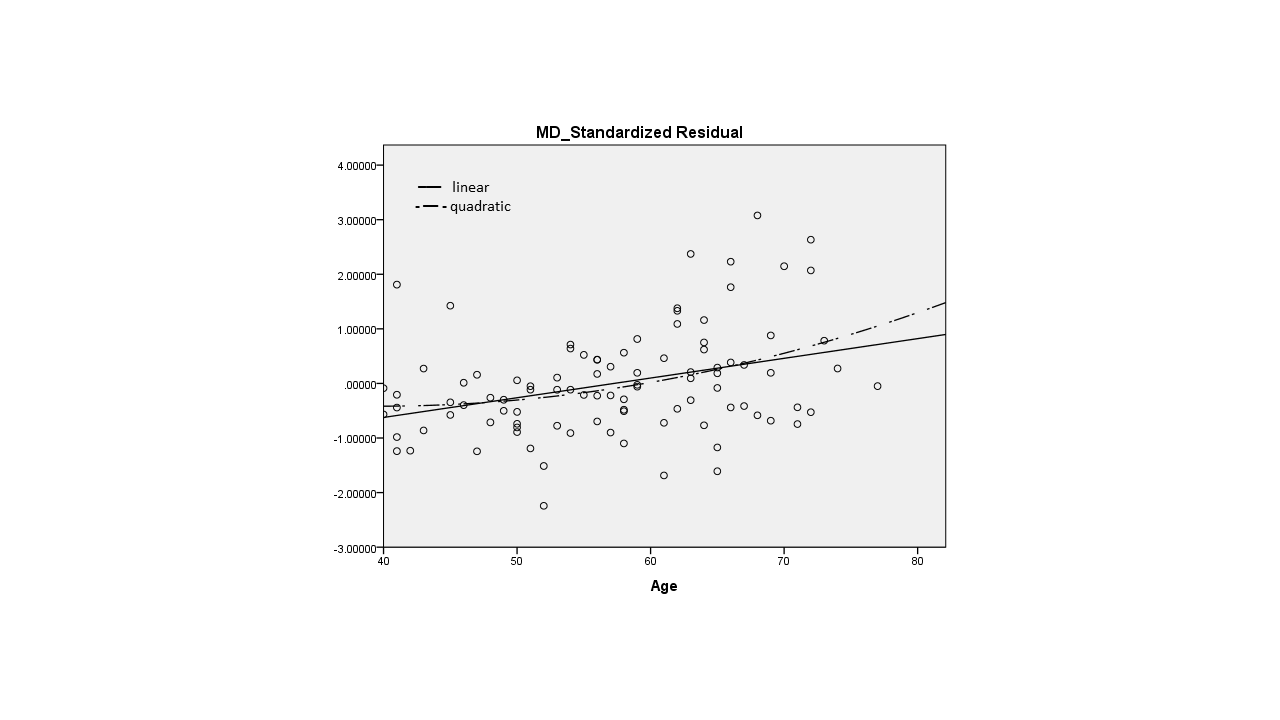

Supplement: S4 Fig — (TIF) [file pone.0205718.s004.tif]
